# Cases Network: we need you for the next stage

**DOI:** 10.1186/1757-1626-2-6366

**Published:** 2009-03-16

**Authors:** Richard Smith

**Affiliations:** 1 Cases Journal, Middlesex House, 34-42 Cleveland Street, London W1T 4LB, UK

## 

Until recently *Cases Journal* and the *Journal of Medical Case Reports* were part of BioMed Central, which, as many readers have noticed, has been sold to Springer Science+Business Media. *Cases Journal* and the *Journal of Medical Case Reports* have not, however, been sold. Instead they will join together with Cases Database to form Cases Network. Both of these developments are good news for science and medicine.

The sale of BioMed Central to Springer Science+Business Media is good news because it both demonstrates how the open access model can work in business terms and brings open access into mainstream science publishing. It's still my belief, as it has been for more than a decade, that all science will be open access eventually, and the sale was an important step. I've been talking recently to an academic colleague who has moved from a university in London to a position in pubic health research in India. Suddenly he struggles to access information. He is denied one of the fundamental tools of his trade, and it's immoral that practitioners and hence their patients and populations should be so denied when almost all of the information he needs has been funded with public money. Traditional publishers are denying access to life saving information.

The sale is also good news for Cases Network because it brings resources for investment and means that the senior managers can concentrate on developing the network. We can move faster with implementing a vision of achieving the best available collection of case reports.

The *Journal of Medical Case Reports* has already established itself as a high quality journal publishing case reports that are both important and original. Each case published there carries an important message.

Every case published in *Cases Journal* is also important - but in a different way. *Cases Journal* aims to celebrate the extraordinary in the ordinary. When we look closely every case is special and different just as every person is special and every face is different. We want to publish tens of thousands of cases, and we still have to achieve "lift off." We have published over 550 cases since we launched in May, but we need to increase that at least ten fold. Our challenge, which I relish, is to change a medical mindset that says that writing and publishing are for "pointy heads" not for ordinary practitioners.

"Ordinary" practitioners (and soon we may ban the word ordinary because closely examined nothing is ordinary) should write and publish for two interlocked reasons. Firstly, they benefit themselves. Writing opens up thoughts you never knew you had and pushes you towards precision. As the surgeon Atul Gawande writes in his marvelous book *Better*: "For all its complexity…Because medicine is a retail enterprise, because doctors provide their services to one person after another, it can be a grind. You can lose your larger sense of purpose, but writing lets you step back and think through a problem. Even the angriest rant forces the writer to achieve a degree of thoughtfulness."[1]

But more importantly what you may think of as ordinary or even boring can be contributed to the database and become part of something very valuable. Gawande quotes Lewis Thomas, the great biologist, quoting John Ziman, the great physicist, (all this quoting is getting ridiculous but it's worth it) as saying: "The invention of a mechanism for the systematic publication of 'fragments' of scientific work may well have been the key event in the history of modern science." The development of Web 2.0 allows the publication of fragments, case histories, in a way that was never possible before. You could argue that as a practitioner you have a duty not just to treat your individual patients but also to contribute to the broader whole.

Because the third part of the Cases Network will be a database of cases with a highly sophisticated search engine that will allow practitioners to find cases very similar to those that they are treating and to discover what happened to them. Guidelines, as we know, are poor at giving guidance on patients with comorbidities, which is the everyday stuff of medicine, and accessing the database will give invaluable information. [2] What might be the best thing for an 80-year-old obese woman with hypertension, osteoarthritis, depression, and mild dementia?

If "ordinary" doctors will submit "ordinary" cases then we can develop the raw material for a hugely useful database. As Enzo Grossi has explained in an editorial in *Cases Journal*, [3] it is no easy thing to build a sophisticated search engine - but we are well on the way to doing it. I urge you to do your bit and contribute cases. My bet is that you'll learn to love writing up and submitting cases once you get started.
